# Organic food use, meat intake, and prevalence of gestational diabetes: KOALA birth cohort study

**DOI:** 10.1007/s00394-021-02601-4

**Published:** 2021-06-05

**Authors:** Ana Paula Simões-Wüst, Carolina Moltó-Puigmartí, Martien C. J. M. van Dongen, Carel Thijs

**Affiliations:** 1 Research Department, Clinic Arlesheim, Arlesheim, Switzerland; 2 Department of Obstetrics, Zurich University Hospital, Schmelzbergstrasse 12/PF 125, Path G51a, 8091 Zurich, Switzerland; 3 Department of Epidemiology, CAPHRI Care and Public Health Research Institute, Maastricht University, Maastricht, The Netherlands

**Keywords:** Gestational diabetes, Organic food, Meat consumption, Diet composition, Food patterns

## Abstract

**Purpose:**

To evaluate whether consumption of organic food and reduced intake of meat products in pregnancy are associated with lower prevalence of gestational diabetes (GD).

**Methods:**

Women participating in the KOALA Birth Cohort Study with valid informed consent, a singleton pregnancy and information on their food intake were considered in this cross-sectional analysis. Participants with and without GD were compared with each other in terms of dietary characteristics (*n* = 37 and *n* = 2766, respectively). Multivariable logistic regression (LR) was used to adjust for relevant covariates.

**Results:**

Organic food consumption tended to be lower, although not significantly, in women with GD compared to women without GD, whereas consumption of meat was positively associated with GD prevalence. LR modelling showed that GD was significantly associated with higher consumption of meat and, in addition, also of cheese, after adjustment for other relevant covariates. GD was associated with some indicators of animal product intake, namely dietary animal to plant protein ratio and maternal plasma arachidonic acid (for the latter, data available for *n* = 16 and *n* = 1304, respectively). Food patterns of participants with GD were characterised by more meat products and less vegetarian products.

**Conclusions:**

Due to the low number of participants with GD, results have to be interpreted cautiously. Consumption of organic food during pregnancy does not seem to be markedly associated with a lower GD prevalence; lower intake of meat and cheese, irrespective of its origin (organic or conventional), does. The latter supports previous studies suggesting a causal association between consumption of animal products and GD.

**Supplementary Information:**

The online version contains supplementary material available at 10.1007/s00394-021-02601-4.

## Introduction

Gestational diabetes (i.e. diabetes with first diagnosis or onset during pregnancy, GD) accounts for most cases of diabetes during pregnancy and is one of the most common pregnancy-related disorders. GD-associated perinatal complications include macrosomia, hypoglycaemia, respiratory distress syndrome, polycythaemia, hyperbilirubinemia, cardiomyopathy and infant death. In the last years, implications of in utero exposure to maternal GD for offspring long-term health have been unveiled. Research shows that GD can be associated with future adiposity, adverse cardiometabolic outcomes and reduced cognitive ability [1–3]. GD prevalence has been increasing in the last decade and at present, it occurs in approximately seven percent of all pregnancies [4]. This increase accompanies the worldwide rise in type 2 diabetes prevalence, which is likely to be related to a rapid replacement of traditional diets by dietary patterns with high proportions of animal products, processed industrial foods, refined sugars, saturated fats and oils [5–7]. There are some indications that dietary patterns can influence GD development as well. Dietary patterns characterised by high amounts of whole grains, fruits, and vegetables seem to reduce the GD risk, and Western dietary patterns as well as those characterised among others by high amounts of (red and/or processed) meat are associated with higher prevalence [8–10]. Currently, high meat consumption deserves special attention, because evidence for a direct negative impact on human health is accumulating [11–15] and meat production practices are unsustainable [16]. Discussions about how much meat should be consumed and how to produce meat products in a sustainable way are needed [17].

Production of organic foods relies on ecological processes, biodiversity, and cycles adapted to local conditions. At the same time, agricultural inputs with unknown or adverse effects are avoided. These include genetically modified seeds, synthetic fertilisers and pesticides, preventive veterinary drugs, and most preservatives, flavour enhancers and other additives, and irradiation during processing [16]. There are still diverging opinions about sustainability of organic agriculture [18, 19] and the impact of consumption of organic food on health (reviewed in [20]). A few studies suggest the presence of higher amounts of health-promoting substances, such as favourable fatty acids and vitamins, in organic food (see [20] and references therein). The lower use of synthetic fertilisers and pesticides, veterinary drugs with preventive objectives, and preservatives and additives is per se promising. Recent work reveals that—likely due to the low levels of synthetic pesticides—consumption of organic food is inversely associated with the risk of type 2 diabetes [21].

Several studies were initiated to investigate health-related effects of organic food consumption during pregnancy, a period in which health expectations are particularly relevant, since they can long-term affect both mothers’ and their offspring’s health. Results suggest favourable impacts of organic food consumption on the prevalence of preeclampsia [22] and child hypospadias [23], as well as on the later development of child atopic sensitisation, allergies and eczemas [24–26]. Moreover, our previous work with data from the Dutch KOALA-study revealed that organic food consumption is associated with lower prevalence of maternal overweight and adiposity during pregnancy [27], which suggests a beneficial effect of food of organic production on GD development. Although the results obtained with the subgroup of KOALA-participants for which additional health-related biomarkers are available were in line with this hypothesis [28], the sample size was not big enough for its definitive verification.

Analyses of European cohort studies from France [29], Germany [30], Norway [31], and the Netherlands [27] on organic food consumption are unanimous in showing that intake of organic food goes hand in hand with lower consumption of meat and meat products and with specific food patterns. Therefore, it is highly challenging to try to disentangle whether associations between organic food consumption and health-related outcomes can be truly attributed to the organic food by itself, or otherwise to the dietary composition or overall food patterns that are associated with the consumption of organic food. In our previous study, we showed that part of—but not all—the observed associations between organic food consumption and health-related biomarkers could be explained by the food patterns accompanying the consumption of organic food [28]. Following this idea, and using data from the whole KOALA-cohort, we now aimed at investigating the associations between (i) the consumption of organic food, (ii) the consumption of meat and meat products, (iii) dietary composition, (iv) dietary indicators and an independent blood plasma indicator of animal product intake, and (v) food patterns, and the development of GD.

## Methods

### Study design and participants

Recruitment of healthy, pregnant women took place from October 2000 onwards, at midwives’ practices in the southern part of the Netherlands (*n* = 2434). They were participating in an ongoing prospective cohort study on Pregnancy-Related Pelvic Girdle Pain. This study did not use lifestyle-related inclusion criteria and the participants showed a good comparability with the general population [32]. This group is referred to as the general population recruitment group. To enrich the KOALA-study with participants adhering to an alternative lifestyle, 491 pregnant women were recruited from October 2001 onwards through various specific channels (alternative recruitment group). Those specific channels included organic food shops, anthroposophic general practitioners, midwives, and under-five clinics, Rudolf Steiner schools and advertisements in magazines on organic food and other relevant magazines. Recruitment of both groups continued to end of 2002. All women participating in the KOALA-study were enrolled between 14 and 18 weeks of gestation and received detailed questionnaires on sociodemographic and health-related characteristics at the time of recruitment as well as at 30 and 34 weeks of pregnancy (*n* = 2993). The questionnaire issued at 34 weeks of pregnancy dealt with lifestyle and dietary habits during the previous month, including items on organic food purchase and consumption, and a food frequency questionnaire (FFQ).

The population flow chart showing the stepwise recruitment of the study sample can be found in the Supplementary information (Supplementary material 1). Among the 2993 enrolled pregnant women with valid informed consent, 37 had a twin pregnancy, leaving 2956 singleton pregnancies. From these, complete dietary data (FFQ) at week 34 were available for 2821 women; 135 women with no or incomplete dietary data had to be excluded. Also excluded were 14 women whose children turned out to have Down’s syndrome (*n* = 7), or any other congenital disorder (*n* = 7). This means that 2807 women with a singleton pregnancy, available FFQ data and a child without a diagnosed congenital disease were left for further processing. Of the considered 2807 women, 2333 have been recruited from the conventional lifestyle group and 474 from the alternative lifestyle recruitment group.

The primary outcome was gestational diabetes (GD). GD was defined as self-reported gestational diabetes at week 34 (i.e. reported diabetes and absence of diabetes before pregnancy) and/or diagnosis of GD mentioned in the midwife reports. Information on GD status (both self-reported and in midwife reports) was missing in 4 women and 3 of the women without GD had been diagnosed with diabetes mellitus before the current pregnancy and were therefore not at risk of developing GD. This gave rise to exclude another 7 women from the multivariate LR, which was therefore performed with data from 2800 pregnant women.

In all women recruited from January 2002 onwards, blood samples were collected at 34–36 weeks of pregnancy and plasma fatty acids were measured by gas chromatography as described in detail elsewhere [28]. In the present analysis, plasma value of arachidonic fatty acid was included as an independent indicator of animal product intake. These data were available for 1320 (47.1%) of the 2800 women in the analysis.

### Dietary measurements

From the week 34 questionnaire and as described in detail elsewhere [27], answers to the questions dealing with the following topics were used:

1. Origin (conventional or organic) of products from the following seven food groups consumed during the previous month: meat; eggs; vegetables; fruit; milk and milk products; bread; and dried foods (dried legumes and cereals). For each of these food groups, we asked participants to report the percentage of purchased products from organic origin: less than 50%, between 50 and 90%, or more than 90%.
2. Habitual food intake. Intake of food products was assessed based on a 198-item FFQ that is an extended version of a previously existing, validated FFQ [33]. For each of the inquired food products the frequency of use (nine categories: no use, 1–3 days per month, and 1, 2, 3, 4, 5, 6 and 7 days per week) during the previous month had to be recalled and noted down. In addition, the average amount per consumption day was asked for. Depending on the food product this could be done in grams (e.g., grams of meat), natural units (e.g., number of apples, slices of bread), or household units (e.g., glasses of milk, spoons of rice). All food products were classified according to the Dutch Food Consumption Table aggregate level 1 (NEVO-1 food groups). Food products within the same food level 1-group were taken together to obtain the daily intake of each group per participant (in g/day; see [27] for further details). Relative frequency of use of food subtypes was asked for part of the foods.
3. Energy intake, micronutrients and macronutrients. The average daily dietary intake of energy (kJ), micronutrients (mg, µg) and macronutrients (g) was calculated by combining for each FFQ item the frequency and amount data with standard portion sizes (grams) and with nutrient composition per 100 g of food as documented in the 2010 online version of the Dutch Food Composition Table for about 170 nutrients (Voedingscentrum 2010); as in previous work [27], supplements were not considered in this calculation. In the present analysis, energy intake and the data required to calculate dietary ratios haem to non-haem iron and animal to plant protein (indicators of animal product intake) were considered.

### Definition of subgroups of organic food intake and meat intake

As in our previous work [27], the use of organic food was defined based on the questions concerning the origin of the seven food groups mentioned above (meat; eggs; vegetables; fruit; milk and milk products; bread; and dried foods) and on the % organic origin of these seven food groups taken together. The following subgroups were defined according to the consumption of organic food: “ < 50% organic”, if some food groups were of organic origin, but not all were reported as being more than 50% organic; “ > 50% organic”, if in all consumed food groups at least 50% was of organic origin [27]. While doing so, only the consumed food groups were considered (a few participants reported not consuming a specific food group, e.g. meat). The remaining group of participants who used all consumed food groups mostly of conventional origin was considered as the “conventional” group.

Regarding meat intake, participants were categorised in quartiles according to their consumption of meat, processed meat and poultry (food group 23 from the 2010 NEVO-1 classification).

### Statistical methods

Differences in participant characteristics between the groups without (reference) and with GD were evaluated by analysis of variance (ANOVA) and chi-squared test for continuous and categorical variables, respectively.

To identify NEVO-1 food groups associated with GD, multivariable logistic regression was used with GD as the outcome variable, and a stepwise backward elimination of NEVO-1 food groups, using an F-to-enter (PIN) of 0.05 and a F-to-remove (POUT) of 0.10 as the criteria to include or remove a variable in each consecutive step.

Multivariable logistic regression analysis was used to estimate the strength of the association of organic food use and meat intake, with GD status as the outcome, adjusting in first instance only for maternal constitutional factors (maternal age, gravidity). To adjust for possible confounding by (other) food groups, we first identified NEVO-1 food groups associated with GD (see above). These food groups were added to the initial models of organic food and meat intake, respectively. As a final step, other potential confounding covariates were added to derive the final, fully adjusted model, including: maternal age, gravidity, organic food consumption, maternal smoking in pregnancy, living area, season of filling out the questionnaire, and recruitment group and period (to adjust for secular trend: conventional group recruited from October 2000 to October 2001; conventional group recruited from October 2001 to end of 2002; alternative group recruited from October 2001 to end of 2002).

To evaluate possible influences of energy intake and of pre-pregnancy BMI, we used sensitivity analyses by comparing the final model with and without total energy intake and with and without pre-pregnancy BMI. Additional logistic regression analyses were used to estimate odds ratios for the association between selected indicators of animal product intake (dietary ratios haem to non-haem iron and animal to plant protein and plasma value of arachidonic fatty acid) and GD, with and without adjustment for the covariates shown in table footnotes.

Multivariable analysis was performed to calculate values that could be used to substitute missing values for any of the food items (*n* = 15, one food item missing per participant). Having imputed the calculated values, Principal Components Analysis (PCA) of all food items was performed to further characterise food patterns. After setting the threshold on 0.15, the varimax rotation with Kaiser-normalisation led to the following results. The Kaiser–Meyer–Olkin measure verified the sample adequacy for analysis (KMO = 0.698) and the Bartlett’s test of sphericity Χ2 (22′578) = 97′741.962, *p* = 0.000, indicated that correlations between items were sufficiently high for PCA.

Data were analysed with IBM^®^ SPSS^®^ Statistics Version 19, except the logistic regression analyses, for which Version 25 was used. Statistical significance was set at *p* ≤ 0.05.

## Results

### Main participant characteristics

Women with and without GD were comparable in terms of recruitment characteristics, maternal age, level of education and living area (Table 1). In comparison with women without GD (reference group), women with GD had a slightly shorter pregnancy duration (mean ± standard deviation of 39.0 ± 1.4 weeks versus 39.5 ± 1.5 weeks) and higher average gravidity (2.3 ± 1.7 versus 1.9 ± 1.0). Clear-cut differences were observed with regard to mothers’ BMI before pregnancy (27.3 ± 5.8 versus 23.6 ± 3.9). Energy intake was similar in the two groups.

**Table 1:** Comparison of characteristics of participants with and without gestational diabetes (*n* = 2803), expressed as % or mean ± SD

Characteristic	With gestational diabetes (*n* = 37)		Without gestational diabetes (*n* = 2766)		*p* value
	*n* or (mis)	mean ± SD or %	*n* or (mis)	mean ± SD or %	
Recruitment group and period					0.15*
General recruitment group/early period	13	35.1	1227	44.4	
Alternative recruitment group/late period	4	10.8	470	17.0	
General recruitment group /late period	20	54.1	1069	38.6	
Age of the mother at delivery (years)	37	32.24 ± 3.9	2766	31.5 ± 4.1	0.24
Pregnancy duration (weeks)	36	39.0 ± 1.4	2754	39.5 ± 1.5	**0.04**
Missings	1		12		
Highest education					0.15
Lower	6	16.2	287	10.4	
Middle	20	54.1	1050	38.0	
Higher	7	18.9	929	33.6	
Academic	4	10.8	374	13.5	
Others	0	0.00	126	4.6	
Gravidity	37	2.3 ± 1.7	2766	1.9 ± 1.0	**0.02**
Living area (province)					0.43
South (Limburg)	14	37.8	1128	40.8	
Other	23	62.2	1638	59.2	
Mothers’ BMI before pregnancy	35	27.3 ± 5.8	2700	23.6 ± 3.9	** < 0.001**
Missings	2		66		
Energy intake at gestational week 34 (kJ/day)	37	10,055 ± 1931	2766	10,591 ± 2586	0.21

Statistically significant *p*-values are written in bold

*p = 0.095 if only the late period subgroups are considered

### Consumption of organic food and meat intake in relation to gestational diabetes status

When the consumption of organic products in participants with and without GD was compared, a trend of lower organic food consumption in GD women was observed, which, however, was not significant (see Table 2). Further adjustments for NEVO-1 food groups and other covariables did not essentially alter these results (not shown).

**Table 2:** Associations between consumption of organic food and gestational diabetes status and between meat intake and gestational diabetes status, expressed as number of cases relative to total (and %; *n* = 2803) and odds ratio obtained in logistic regression models (and 95% CI, *n* = 2800^a)^)

	No. of cases with GD/total (%)	OR (95% CI) unadjusted	OR (95% CI) adjusted ^b^
Organic food consumption			
Conventional	25/1728 (1.5%)	1.00 (reference)	1.00 (reference)
< 50%	10/851 (1.2%)	0.81 (0.39, 1.69)	0.71 (0.32, 1.54)
> 50%	2/224 (0.9%)	0.61 (0.14, 2.61)	0.53 (0.12, 2.30)
Test-for-trend		*p* = 0.42	*p* = 0.26
Meat consumption (quartiles of NEVO-1 group 23)			
Q1 0–74 g/d	3/700 (0.4%)	1.00 (reference)	1.00 (reference)
Q2 74–106 g/d	8/701 (1.1%)	2.69 (0.71, 10.18)	2.74 (0.72, 10.41)
Q3 106–136 g/d	10/702 (1.4%)	3.36 (0.92, 12.25)	3.47 (0.95, 12.72)
Q4 136–368 g/d	16/700 (2.3%)	5.44 (1.58, 18.76)	5.22 (1.48, 18.37)
Test-for-trend		***p* = 0.003**	***p* = 0.006**

Statistically significant *p*-values are written in bold

^a^Participants with diabetes before pregnancy were excluded from the logistic regression models

^b^Covariates included in the model: mother’s age at delivery and gravidity

The proportion of women with GD increased successively across the quartiles of meat intake (NEVO-1 group 23), with a consistent dose–response relationship towards higher GD risk associated with higher meat intake (test-for-trend over the quartiles *p* = 0.006, initial adjusted model, Table 2). Among the participants in the fourth quartile, with the highest meat consumption (136–367 g/d), the prevalence of GD was of 16 out of 700, i.e. 2.3%, whereas the prevalence was only 3 out of 700, i.e. 0.4%, among the participants in the first quartile.

### Dietary composition versus gestational diabetes

Following an explorative approach, we investigated the association between the intake of products from various food groups and GD status. Since intakes from various NEVO-1 food groups are often inter-related, we initially considered to include all food groups—not only meat—in the multivariable analysis. A stepwise elimination resulted in the retention of six food groups in the final model. NEVO-1 food groups positively associated with GD were: meat/meat products/poultry (group 23), cheese (group 11) and drinks (group 2), whereas intake of sugar/sweets/sweet sauces (group 20), cereals/cereal products (group 8), and pastry and biscuits (group 7) were negatively associated with GD status (Table 3, unadjusted). These results were in most cases confirmed by a comparison of the diet composition in terms of NEVO-1 groups between participants with and without GD (Supplementary material 2), that also revealed higher consumption of eggs (group 5, borderline significance). In the final, fully adjusted model, both meat and cheese intake remained strongly associated with GD risk. The odds for GD was 2.67 (95%CI 1.14, 6.26) higher with each 100 g/day increase of meat intake, and 5.68 (1.54, 20.9) for each 100 g/day increase of cheese intake, independent from each other and from the intake of the other food groups (Table 3, adjusted). Total energy intake was not associated with GD (Table 3, adjusted), and the results for meat and cheese did not change when energy intake was eliminated from this model (results not shown). Finally, adding the pre-pregnancy BMI to this model, only slightly attenuated the odds for GD in the case of meat (from 2.67 to 2.41; *p* values 0.02 and 0.04) and even slightly increased them in the case of cheese (from 5.68 to 5.95; *p* value 0.01 in both cases).

**Table 3:** Associations between consumption of products from the most relevant food groups (100 g of each) and gestational diabetes status as estimated by unadjusted and adjusted logistic regression models (odds ratios and 95% confidence intervals; *n* = 2800)

Characteristic	OR (95%CI) unadjusted	*p* value	OR (95%CI) adjusted^a^	*p* value
Group 23, meat/meat products/poultry	2.19 (1.2, 4.00)	0.01	2.67 (1.14, 6.26)	**0.02**
Group 11, cheese	3.87 (1.26, 11.8)	0.02	5.68 (1.54, 20.9)	**0.01**
Group 2, drinks	1.06 (1.01, 1.10)	0.01	1.05 (0.99, 1.12)	0.12
Group 20, sugar/sweets/sweet sauces	0.47 (0.24, 0.91)	0.03	0.69 (0.31, 1.55)	0.37
Group 8, cereals/cereal products	0.31 (0.11, 0.90)	0.03	0.33 (0.10, 1.13)	0.08
Group 7, pastry and biscuits	0.24 (0.05, 1.14)	0.07	0.31 (0.06, 1.63)	0.17
Energy intake (100 kJ/day)	–	–	0.99 (0.96, 1.01)	0.24

Participants with diabetes before pregnancy were excluded from the logistic regression models

Statistically significant *p*-values are written in bold

^a^Covariates included in the model: energy intake, mother’s age at delivery, gravidity, recruitment group, organic food consumption, smoking, living area and season of filling out the questionnaire

### Dietary and independent blood plasma indicators of animal product intake

We reasoned that the participants who consumed higher amounts of meat/meat products/poultry and cheese (NEVO-1 groups 23 and 11) would exhibit higher values of three indicators of animal product intake: dietary ratios haem to non-haem iron and animal to plant protein, and maternal plasma arachidonic acid levels [34]. The correlations between these indicators and the consumption of products from NEVO-1 groups 23 and 11 were investigated by calculating Pearson correlation coefficients (Supplementary material 3). The haem to non-haem iron ratio, and the animal to non-animal protein ratio showed reasonable correlations with meat intake (correlation coefficients 0.62 and 0.53, respectively), with cheese intake (0.12 and 0.53) and with each other (0.53). Maternal plasma arachidonic acid was weakly but significantly associated with meat and cheese intake (correlation coefficients 0.14 and 0.08). The haem to non-haem iron ratio and the animal to non-animal protein ratio were higher in the participants with GD (Supplementary material 4); both dietary indicators were positively associated with GD risk (although the first one lost the borderline statistical significance when adjusting for maternal age and gravidity; Table 4). Maternal plasma arachidonic acid was strongly associated with GD risk (*p* = 0.01, both unadjusted and adjusted, Table 4).

**Table 4:** Associations between selected indicators for animal product intake and gestational diabetes status as estimated by unadjusted and adjusted logistic regression modelling (odds ratios and 95% confidence intervals; *n* = 2800)

Characteristic	OR (95%CI) unadjusted	*p* value	OR (95%CI) adjusted^a^	*p* value
Ratio haem to non-haem iron (maternal diet; × 1/10)	1.80 (1.00, 3.23)	0.05	1.61 (0.84, 3.07)	0.15
Ratio animal to plant protein (maternal diet)	1.77 (1.14, 2.73)	**0.01**	1.78 (1.12, 2.82)	**0.02**
Arachidonic acid (maternal plasma; 1% wt)^b^	1.72 (1.18, 2.51)	**0.01**	1.72 (1.17, 2.52)	**0.01**

Participants with diabetes before pregnancy were excluded from the logistic regression models

Statistically significant *p*-values are written in bold

^a^Covariates included in the model: mother’s age at delivery and gravidity

^b^Data available for 1304 pregnant women without GD and 16 with GD

### Food patterns versus gestational diabetes

Finally, we wanted to gain a more real insight into the everyday diet composition of the participants with and without GD. In the PCA performed to characterise food patterns, seven components were found (with eigenvalues over Kaiser’s criterion higher than 2; supported by the scree plot, not shown) that together explained 13.2% of the food items variance. Based on the underlying constituents, we labelled these main components as follows: “lacto-ovo-vegetarian”, “fast food”, “raw vegetables salad”, “fish”, “cooked vegetables”, “Italian-like kitchen and sweet”, and “meat” (see corresponding food items described in the order of decreasing absolute factor loadings in Supplementary material 5, both for positive and for negative loadings). The scores for “meat” were higher in women with GD than in those without, whereas scores for “lacto-ovo-vegetarian” and “Italian-like kitchen and sweet” were lower (Supplementary material 6). Taken together, participants with GD consumed less plant-based products (e.g., tofu, soya, quorn, muesli, legumes, grains, vegetables as pumpkin and fennel, seeds, sunflower/pumpkin seeds), more meat and meat products (e.g., poultry, pork tenderloin, fricandeau, schnitzel, sausages, minced meat, ham), and more products such as cheese, French fries/chips, peanuts, cocktail nuts, chocolates, pralines, cake, tart, waffles, biscuits and cookies.

## Discussion

Our results show that, while consumption of organic food does not seem to markedly affect GD prevalence, higher intake of meat and meat products is associated with a higher prevalence. Based on logistic regression modelling, a direct dependency between consumption of meat products and GD prevalence was found. Additional LR models, in which the most relevant food groups were included, confirmed that the risk of GD is statistically significantly associated with higher consumption of meat, even after adjustment for confounders. In addition, we found that cheese consumption is associated with GD status as well, independently from meat intake. Moreover, GD is associated with dietary (haem to non-haem iron ratio, animal to plant protein ratio) and plasma (arachidonic acid levels) indicators of animal product intake. Finally, these findings were corroborated with a PCA analysis, which showed that food patterns of GD participants are characterised by higher meat product intake and lower vegetarian product intake.

A major strength of the present work is the combination of various approaches to investigate associations between diet characteristics and GD. A high contrast of participants’ diet composition, the very detailed FFQ used in the KOALA study, and the availability of data on macronutrients and micronutrients and (for some participants) on blood plasma biomarkers enabled that combination. Based on the nutrients’ data, two dietary indicators of animal product intake could be calculated. Among the plasma biomarkers, the semi-essential fatty acid arachidonic acid has the advantages that its main dietary source are animal products [34, 35], at the same time that, in humans, the ingested amounts of the precursor linoleic acid hardly affect its plasma levels [36]. A major limitation of this analysis is the modest number of women with GD (*n* = 37 for diet characteristics) and hence a limited statistical power to prove or rule out a modest effect of organic food use, although for meat intake the associations appeared strong enough to reach statistical significance even with the limited number of cases. In most cases of GD (20 out of 37), the diagnosis was supported by midwife reports, however in 17 cases, the diagnosis was self-reported only, which can be seen as a study limitation. Another limitation is that the pregnant women filled out the FFQ at pregnancy week 34, i.e. when a GD diagnosis is usually known. Since dietary changes are one of the first measures taken in GD treatment, it is likely that women with GD had already adapted their diet by the time of completing the FFQ. This might explain why—in the unadjusted MLR model—the GD participants seemed to consume lower amounts of sugar/sweets/sweet sauces, cereals, and pastry and biscuits; also, the somewhat higher amounts of drinks ingested might be related to GD-associated thirst. Importantly, increasing meat and cheese consumption is not pursued by GD treatment, so it is not likely that our results on meat and cheese intake are due to reverse causation.

Due to the low number of participants with GD, our results have to be interpreted cautiously. Nevertheless, they suggest that GD prevalence could be lower if women would reduce their meat and cheese intake. If the effect of meat consumption on GD risk would be causal, the effect size in this study (OR 2.83 per 100 g/day) could be interpreted as a two to three times lower risk of GD with lowering meat intake from 150 g/day to 50 g/day (i.e. from approximately the population mean plus 1 SD to the population mean minus 1 SD; compare with Supplementary material 2); or as a five times lower risk for pregnant women in the lowest quartile of meat consumption compared to women in the highest quartile (OR 5.22). For cheese intake, the effect size was found to be in the same order of magnitude (OR 5.14 per 100 g extra cheese per day). Our results on meat and cheese are in line with studies showing that Mediterranean Diet, that comprises few animal products, reduces GD incidence [37, 38]. Furthermore, they are supported by previous work revealing an association between Western dietary patterns and GD [9]. The nature of such associations reserves further investigations. Seeing that vegetarian diets affect gut microbiota [39], a modulation of gut microbiota by dietary patterns could be an appealing possibility.

The relevant time window to adjust dietary pattern and thereby minimise the risk of GD development cannot be extrapolated from the present analysis. Dietary patterns in the past, i.e. long before the pregnancy, might influence health status at the beginning of pregnancy, e.g. by long-term influencing pre-pregnancy BMI that per se is a risk factor for GD [40]. It is, however, also conceivable that dietary pattern changes during pregnancy might still affect the development of GD. In fact, tailored dietary planning—mostly by reducing sugar-sweetened beverages—is an important part of GD management and has the immediate goal of improving glycaemic control [41]. Interestingly, reducing hypercaloric food product intake—including meat products and cheese—may also contribute to energy restriction by obese pregnant women and prevent excessive weight gain during pregnancy together with its long-term consequences ([40, 42]). This is in line with very recent work suggesting that vegetarian diets during pregnancy are associated with reduced gestational weight gain [43].

GD is known to be associated with marked perinatal complications and with adiposity, adverse cardiometabolic outcomes and reduced cognitive ability of the offspring. Our work suggests that pre-conceptional and early pregnancy dietary advice should include recommendations to partially replace meat, meat products and cheese by plant-based products at the earliest opportunity in an attempt to minimise the risk of developing GD.

## Supplementary Information

Below is the link to the electronic supplementary material.

Supplementary file1 (DOCX 63 KB)

## Data Availability

All data generated or analysed during the current study are available from the corresponding author on reasonable request.

## Abbreviations

BMI: Body mass index
FFQ: Food frequency questionnaire
GD: Gestational diabetes
KOALA: ‘Kind, Ouders en Gezondheid: Aandacht voor Leefstijl en Aanleg’ (child, parents and health, addressing lifestyle and constitution)
LR: Logistic regression
NEVO-1: Dutch food consumption table aggregate level 1
PCA: Principal component analysis

## References

[1] Fraser A, Lawlor DA (2014). Long-term health outcomes in offspring born to women with diabetes in pregnancy. Curr Diab Rep.

[2] Damm P, Houshmand-Oeregaard A, Kelstrup L, Lauenborg J, Mathiesen ER, Clausen TD (2016). Gestational diabetes mellitus and long-term consequences for mother and offspring: a view from Denmark. Diabetologia.

[3] Farahvar S, Walfisch A, Sheiner E (2019). Gestational diabetes risk factors and long-term consequences for both mother and offspring: a literature review. Expert Rev Endocrinol Metab.

[4] Reece EA, Leguizamon G, Wiznitzer A (2009). Gestational diabetes: the need for a common ground. Lancet.

[5] Tilman D, Clark M (2014). Global diets link environmental sustainability and human health. Nature.

[6] Drewnowski A, Popkin BM (1997). The nutrition transition: new trends in the global diet. Nutr Rev.

[7] Tucker LA, Erickson A, LeCheminant JD, Bailey BW (2015). Dairy consumption and insulin resistance: the role of body fat, physical activity, and energy intake. J Diabetes Res.

[8] Hassani Zadeh S, Boffetta P, Hosseinzadeh M (2020). Dietary patterns and risk of gestational diabetes mellitus: a systematic review and meta-analysis of cohort studies. Clin Nutr ESPEN.

[9] Quan W, Zeng M, Jiao Y, Li Y, Xue C, Liu G, Wang Z, Qin F, He Z, Chen J (2021). Western dietary patterns, foods, and risk of gestational diabetes mellitus: a systematic review and meta-analysis of prospective cohort studies. Adv Nutr.

[10] Roustazadeh A, Mir H, Jafarirad S, Mogharab F, Hosseini SA, Abdoli A, Erfanian S (2021). A dietary pattern rich in fruits and dairy products is inversely associated to gestational diabetes: a case-control study in Iran. BMC Endocr Disord.

[11] Pan A, Sun Q, Bernstein AM, Schulze MB, Manson JE, Stampfer MJ, Willett WC, Hu FB (2012). Red meat consumption and mortality: results from 2 prospective cohort studies. Arch Intern Med.

[12] Barnard N, Levin S, Trapp C (2014). Meat consumption as a risk factor for type 2 diabetes. Nutrients.

[13] Papier K, Fensom GK, Knuppel A, Appleby PN, Tong TYN, Schmidt JA, Travis RC, Key TJ, Perez-Cornago A (2021). Meat consumption and risk of 25 common conditions: outcome-wide analyses in 475,000 men and women in the UK Biobank study. BMC Med.

[14] Ibsen DB, Steur M, Imamura F, Overvad K, Schulze MB, Bendinelli B, Guevara M, Agudo A, Amiano P, Aune D (2020). Replacement of red and processed meat with other food sources of protein and the risk of type 2 diabetes in european populations: the EPIC-interact study. Diabetes Care.

[15] Alshahrani SM, Fraser GE, Sabate J, Knutsen R, Shavlik D, Mashchak A, Lloren JI, Orlich MJ (2019). Red and processed meat and mortality in a low meat intake population. Nutrients.

[16] Strassner C, Cavoski I, Di Cagno R, Kahl J, Kesse-Guyot E, Lairon D, Lampkin N, Loes AK, Matt D, Niggli U (2015). How the organic food system supports sustainable diets and translates these into practice. Front Nutr.

[17] Simões-Wüst AP, Dagnelie PC (2019). To be or not to be for humankind—organic diets revisited for a sustainable development. Sustain Earth.

[18] Lee KS, Choe YC, Park SH (2015). Measuring the environmental effects of organic farming: a meta-analysis of structural variables in empirical research. J Environ Manag.

[19] Meier MS, Stoessel F, Jungbluth N, Juraske R, Schader C, Stolze M (2015). Environmental impacts of organic and conventional agricultural products–are the differences captured by life cycle assessment?. J Environ Manag.

[20] Brantsaeter AL, Ydersbond TA, Hoppin JA, Haugen M, Meltzer HM (2017). Organic food in the diet: exposure and health implications. Annu Rev Public Health.

[21] Kesse-Guyot E, Rebouillat P, Payrastre L, Alles B, Fezeu LK, Druesne-Pecollo N, Srour B, Bao W, Touvier M, Galan P (2020). Prospective association between organic food consumption and the risk of type 2 diabetes: findings from the NutriNet-Sante cohort study. Int J Behav Nutr Phys Act.

[22] Torjusen H, Brantsaeter AL, Haugen M, Alexander J, Bakketeig LS, Lieblein G, Stigum H, Naes T, Swartz J, Holmboe-Ottesen G (2014). Reduced risk of pre-eclampsia with organic vegetable consumption: results from the prospective Norwegian Mother and Child Cohort Study. BMJ Open.

[23] Brantsaeter AL, Torjusen H, Meltzer HM, Papadopoulou E, Hoppin JA, Alexander J, Lieblein G, Roos G, Holten JM, Swartz J (2016). Organic food consumption during pregnancy and hypospadias and cryptorchidism at birth: the Norwegian mother and child cohort study (MoBa). Environ Health Perspect.

[24] Rist L, Mueller A, Barthel C, Snijders B, Jansen M, Simões-Wüst AP, Huber M, Kummeling I, von Mandach U, Steinhart H (2007). Influence of organic diet on the amount of conjugated linoleic acids in breast milk of lactating women in the Netherlands. Br J Nutr.

[25] Thijs C, Muller A, Rist L, Kummeling I, Snijders BE, Huber M, van Ree R, Simões-Wüst AP, Dagnelie PC, van den Brandt PA (2011). Fatty acids in breast milk and development of atopic eczema and allergic sensitisation in infancy. Allergy.

[26] Kummeling I, Thijs C, Huber M, van de Vijver LP, Snijders BE, Penders J, Stelma F, van Ree R, van den Brandt PA, Dagnelie PC (2008). Consumption of organic foods and risk of atopic disease during the first 2 years of life in the Netherlands. Br J Nutr.

[27] Simões-Wüst AP, Molto-Puigmarti C, van Dongen MC, Dagnelie PC, Thijs C (2017). Organic food consumption during pregnancy is associated with different consumer profiles, food patterns and intake: the KOALA birth cohort study. Public Health Nutr.

[28] Simões-Wüst AP, Molto-Puigmarti C, Jansen EH, van Dongen MC, Dagnelie PC, Thijs C (2017). Organic food consumption during pregnancy and its association with health-related characteristics: the KOALA birth cohort study. Public Health Nutr.

[29] Kesse-Guyot E, Peneau S, Mejean C, Szabo de Edelenyi F, Galan P, Hercberg S, Lairon D (2013). Profiles of organic food consumers in a large sample of French adults: results from the Nutrinet-Sante cohort study. PLoS ONE.

[30] Eisinger-Watzl M, Wittig F, Heuer T, Hoffmann I (2015). Customers purchasing organic food—do they live healthier? Results of the German national nutrition survey II. Eur J Nutr Food Saf.

[31] Torjusen H, Brantsaeter AL, Haugen M, Lieblein G, Stigum H, Roos G, Holmboe-Ottesen G, Meltzer HM (2010). Characteristics associated with organic food consumption during pregnancy; data from a large cohort of pregnant women in Norway. BMC Public Health.

[32] Bastiaanssen JM, de Bie RA, Bastiaenen CH, Heuts A, Kroese ME, Essed GG, van den Brandt PA (2005). Etiology and prognosis of pregnancy-related pelvic girdle pain; design of a longitudinal study. BMC Public Health.

[33] Grootenhuis PA, Westenbrink S, Sie CM, de Neeling JN, Kok FJ, Bouter LM (1995). A semiquantitative food frequency questionnaire for use in epidemiologic research among the elderly: validation by comparison with dietary history. J Clin Epidemiol.

[34] Seah JY, Gay GM, Su J, Tai ES, Yuan JM, Koh WP, Ong CN, van Dam RM (2017). Consumption of red meat, but not cooking oils high in polyunsaturated fat, is associated with higher arachidonic acid status in Singapore Chinese adults. Nutrients.

[35] Saini RK, Keum YS (2018). Omega-3 and omega-6 polyunsaturated fatty acids: dietary sources, metabolism, and significance—a review. Life Sci.

[36] Rett BS, Whelan J (2011). Increasing dietary linoleic acid does not increase tissue arachidonic acid content in adults consuming Western-type diets: a systematic review. Nutr Metab (Lond).

[37] Assaf-Balut C, Garcia de la Torre N, Duran A, Fuentes M, Bordiu E, Del Valle L, Familiar C, Ortola A, Jimenez I, Herraiz MA (2017). A Mediterranean diet with additional extra virgin olive oil and pistachios reduces the incidence of gestational diabetes mellitus (GDM): a randomized controlled trial: the St Carlos GDM prevention study. PLoS ONE.

[38] Olmedo-Requena R, Gomez-Fernandez J, Amezcua-Prieto C, Mozas-Moreno J, Khan KS, Jimenez-Moleon JJ (2019). Pre-pregnancy adherence to the mediterranean diet and gestational diabetes mellitus: a case-control study. Nutrients.

[39] Barrett HL, Gomez-Arango LF, Wilkinson SA, McIntyre HD, Callaway LK, Morrison M, Dekker Nitert M (2018). A vegetarian diet is a major determinant of gut microbiota composition in early pregnancy. Nutrients.

[40] Santos S, Voerman E, Amiano P, Barros H, Beilin LJ, Bergstrom A, Charles MA, Chatzi L, Chevrier C, Chrousos GP (2019). Impact of maternal body mass index and gestational weight gain on pregnancy complications: an individual participant data meta-analysis of European North American and Australian cohorts. BJOG.

[41] Durnwald C (2020) Gestational diabetes mellitus: Glycemic control and maternal prognosis. UpToDate. www.uptodate.com. Accessed 27 Aug 2020

[42] Santos S, Eekhout I, Voerman E, Gaillard R, Barros H, Charles MA, Chatzi L, Chevrier C, Chrousos GP, Corpeleijn E (2018). Gestational weight gain charts for different body mass index groups for women in Europe, North America, and Oceania. BMC Med.

[43] Yisahak SF, Hinkle SN, Mumford SL, Li M, Andriessen VC, Grantz KL, Zhang C, Grewal J (2021). Vegetarian diets during pregnancy, and maternal and neonatal outcomes. Int J Epidemiol.

