# Supplementary material for: Organic food use, meat intake, and prevalence of gestational diabetes: KOALA birth cohort study
Source: Eur J Nutr. 2021 Jun 5;60(8):4463–72. doi: 10.1007/s00394-021-02601-4 (PMC8572217; doi:10.1007/s00394-021-02601-4)
Supplement: Supplementary file 1 — Supplementary file1 (DOCX 63 KB) [file 394_2021_2601_MOESM1_ESM.docx]

**SUPPLEMENTARY INFORMATION – SUPPLEMENTARY MATERIALS**

Ana Paula Simões-Wüst ^a,b^, Carolina Moltó-Puigmartí^c^, Martien CJM van Dongen ^c^, and Carel Thijs^c^

**Organic food use, meat intake, and prevalence of gestational diabetes - KOALA Birth Cohort Study**

^a^ Clinic Arlesheim, Research Department, Arlesheim, Switzerland

^b^ Zurich University Hospital, Department of Obstetrics, Zurich, Switzerland

^c^ Maastricht University, Department of Epidemiology, CAPHRI Care and Public Health Research Institute, Maastricht, The Netherlands

**Correspondence**

PD Dr. Ana Paula Simões-Wüst, University Hospital of Zurich, Department of Obstetrics, Schmelzbergstrasse 12/PF 125, Path G51a, 8091 Zurich, Switzerland; phone: +41 44 2555131; Fax: +41 44 2554430; [anapaula.simoes-wuest@usz.ch](mailto:anapaula.simoes-wuest@usz.ch)

ORCID numbers: APSW: 0000-0002-4489-0952, CT: 0000-0001-6646-5458

**Supplementary material 1** Participants flow chart used to obtain sample analysed.


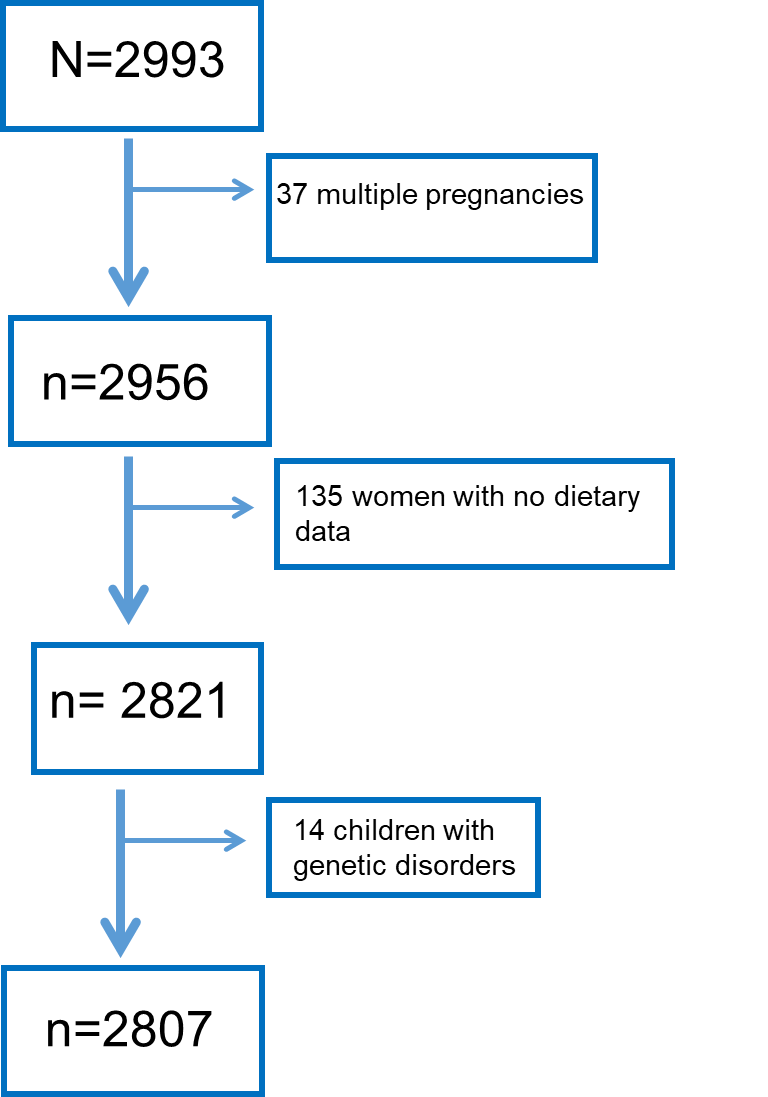


**Supplementary material 2** Comparison of food groups (NEVO groups, level 1) consumption in participants with and without gestational diabetes (n=2803; expressed in g/d and shown as mean ± SD).

|  | **With gestational diabetes**  **(n=37)** | **Without diabetes gestational**  **(n=2766)** | **p-value**  **(ANOVA)** |
| --- | --- | --- | --- |
| **NEVO-1 food groups** | mean ± SD | mean ± SD |  |
| **Group 1, potatoes** | 74.8 ± 46.6 | 70.7 ± 38.0 | 0.52 |
| **Group 2, drinks** | 1500.0 ± 908.4 | 1253.7 ± 524.9 | **0.005** |
| **Group 3, bread** | 184.4 ± 53.4 | 168.0 ± 61.5 | 0.11 |
| **Group 4, miscellaneous** | 0.0 ± 0.2 | 1.2 ±9.5 | 0.45 |
| **Group 5, eggs** | 11.7 ± 9.8 | 9.0 ± 8.3 | 0.05 |
| **Group 6, fruits** | 158.2 ± 75.5 | 149.1 ± 68.8 | 0.43 |
| **Group 7, pastry and biscuits** | 31.0 ± 24.1 | 41.1 ± 28.4 | **0.03** |
| **Group 8, cereals/cereal products** | 47.0 ± 30.9 | 62.1 ± 43.9 | **0.04** |
| **Group 9, vegetables** | 154.6 ± 74.6 | 165.2 ± 97.3 | 0.51 |
| **Group 10, savoury bread spreads** | 2.8 ± 6.5 | 3.9 ± 7.3 | 0.36 |
| **Group 11, cheese** | 30.1 ± 22.1 | 20.7 ± 18.6 | **0.002** |
| **Group 13, milk and milk products** | 445.6 ± 200.2 | 412.9 ± 252.6 | 0.43 |
| **Group 14, nuts, seeds and snacks** | 20.8 ± 18.1 | 22.9 ± 20.8 | 0.54 |
| **Group 15, legumes** | 10.0 ± 9.7 | 9.9 ± 14.1 | 0.95 |
| **Group 17, mixed dishes** | 31.8 ± 28.4 | 38.9 ± 27.7 | 0.12 |
| **Group 18, soups** | 66.7 ± 86.3 | 71.0 ± 75.4 | 0.73 |
| **Group 19, soy vegetarian products** | 1.9 ± 5.6 | 8.9 ±30.8 | 0.17 |
| **Group 20, sugar/sweets/sweet sauces** | 71.1 ± 56.9 | 92.7 ±63.0 | **0.04** |
| **Group 21 fats/oils, /savoury sauces** | 63.2 ± 28.6 | 62.4 ± 27.8 | 0.86 |
| **Group 22, fish** | 40.2 ± 28.5 | 41.6 ± 38.6 | 0.82 |
| **Group 23, meat/meat products/poultry** | 129.8 ± 52.5 | 107.0 ±48.7 | **0.005** |

**Supplementary material 3** Correlations among consumption of NEVO groups 23 (meat/meat products/poultry) and 11 (cheese), and several possible indicators of their consumption, namely dietary ratios of haem to non-haem iron animal to plant protein, and maternal plasma arachidonic acid. Data are shown as Pearson correlation coefficient (significance, N); higher correlations are shaded darker.

|  | **NEVO Group 23, meat/meat products/poultry** | **NEVO Group 11, cheese** | **Ratio haem to non-haem iron (maternal diet)** | **Ratio animal to plant protein (maternal diet)** | **Arachidonic acid (maternal plasma)** |
| --- | --- | --- | --- | --- | --- |
| **NEVO Group 23, meat/meat products/poultry** | 1 (-, 2804) | 0.123 (<0.001, 2804) | 0.623 (<0.001, 2804) | 0.527 (<0.001, 2804) | 0.144 (<0.001, 1326) |
| **NEVO Group 11, cheese** | 0.123 (<0.001, 2804) | 1 (-, 2804) | 0.115 (<0.001, 2804) | 0.527 (<0.001, 2804) | 0.079 (0.004, 1326) |
| **Ratio haem to non-haem iron (maternal diet)** | 0.623 (<0.001, 2804) | 0.115 (<0.001, 2804) | 1 (-, 2804) | 0.527 (<0.001, 2804) | 0.219 (<0.001, 1326) |
| **Ratio animal to plant protein (maternal diet)** | 0.527 (<0.001, 2804) | 0.527 (<0.001, 2804) | 0.527 (<0.001, 2804) | 1 (-, 2804) | 0.183 (<0.001, 1326) |
| **Arachidonic acid (maternal plasma)** | 0.144 (<0.001, 1326) | 0.079 (0.004, 1326) | 0.219 (<0.001, 1326) | 0.183 (<0.001, 1326) | 1 (-,1326) |

**Supplementary material 4** Comparison of dietary ratios of haem to non-haem iron, animal to plant protein, dietary arachidonic acid and maternal plasma arachidonic acid between participants with and without gestational diabetes; expressed as mean ± SD (N).

| **Characteristic** | **With gestational diabetes**  mean ± SD (N) | **Without diabetes gestational**  mean ± SD (N) | **P-value**  **(ANOVA)** |
| --- | --- | --- | --- |
| **Ratio haem to non-haem iron (maternal diet)** | 0.125 ± 0.044 (37) | 0.108 ± 0.051 (2766) | 0.05 |
| **Ratio animal to plant protein (maternal diet)** | 1.862 ± 0.621 (37) | 1.596 ± 0.628 (2766) | **0.01** |
| **Arachidonic acid (maternal plasma, % total fatty acids)** | 8.446 ± 1.486 (16) | 7.5290 ± 1.2713 (1311) | **0.004** |

**Supplementary material 5** Seven main food pattern components as identified by Principal Component Analysis.

**Component 1, “lacto-ovo-vegetarian”**

Positive loadings for tofu, tempeh, soya chunks; vegetarian burger/schnitzel; pumpkin; butter as cooking fat; butter as spread for bread; fennel; sunflower/pumpkin seeds; Tartex and other vegetarian spreads; quorn (small pieces quorn; nut spread, sesame spread; quorn (meat substitute), burger/schnitzel quorn; cheese (as part of a hot meal); grain, buckwheat, bulgur (wheat), couscous, grits; dried or preserved fruit; seitan (seitan); whole milk / full fat yoghurt, biogarde, vifit, etc. without fruit (natural); legumes (kidney beans, white beans, marrowfat beans, lentils, etc.); other nuts (cashew nuts, mixed nuts and raisins, etc.); muesli, cruesli, cereal flakes; Bambu, other coffee substitute; soup as a main course; yeast paste (Marmite, Reformite, etc.); carob spread; carob bars (St.-John's bread, locust bean); soy dessert; other sweet filling (jam, marmalade, honey, (apple-)syrup, etc.); raisin bread, currant bread, muesli bread (cold meal); oatmeal (without milk); coffee milk (liquid coffee creamer) in coffee; whole / full fat milk; porridge with whole full fat (Brinta, oatmeal, semolina, rice, etc.); pancakes (hot meal); (tea, herbal tea); full-cream, full-fat cheese (spread), Gouda, Edam; (unpolished rice); dressing with raw vegetables salad; carrots in raw vegetables salad; other vegetables in raw vegetables salad; lambs lettuce, purslane, water cress in raw vegetables salad; sprouts (e.g., garden cress, mung bean sprouts (taugé), alfalfa) in raw vegetables salad; onions (hot meal); carrots (hot meal); endive (hot meal); kohlrabi (hot meal); vegetarian processed meat products.

Negative loadings for poultry (chicken, turkey); pork tenderloin, fricandeau, schnitzel; luncheon meat sausage, cooked sausage, sausage with smoked bacon-bits (cold meal); loaf fried minced meat (cold meal); low fat / skimmed yoghurt, vifit, etc. with fruit; half fat / semi-skimmed; light margarine as spread on bread, crackers, rusks, etc.; diet margarine as spread on bread, crackers, rusks, etc.; French fries / chips, with hot meal; minced meat hot dog, croquette, or comparable snacks with hot meal; bread rolls, buns (cold meal); minced beef/pork (hot meal); strawberries; bratwurst, lettuce (hot meal); salami sausage, saveloy, luncheon meat (cold meal); cutlet, pork steak, pork collops; boiled ham, fricandeau, cured pork side (cold meal); margarine from packet as cooking fat.

**Component 2, “fast food”**

Positive loadings for pork tenderloin, fricandeau, schnitzel; French fries / chips, with hot meal; French fries (as a snack); salad cream, mayonnaise, etc. (not with raw vegetables); minced meat hot dog, croquette, or comparable snacks with hot meal; white bread (cold meal); fried potatoes (hot meal); solid deep-frying fat as cooking fat; soft drinks (not light), concentrated fruit drink, syrup; canned fruit (apple sauce, rhubarb sauce, pears, etc.); bread rolls, buns (cold meal); gravy prepared from packet; solid frying fat as cooking fat; minced beef/pork (hot meal);

other type of porridge, vanilla custard, pudding; hamburger; ice cream (not lemonade-ice); ready-made pasta meals (macaroni, spaghetti, lasagne, etc.); Chinese noodles (bami), fried rice (nasi), Chinese/Indian food (ready meals); liquid deep-frying fat as cooking; soup as a starter or with bread; yoghurt drink (Yokidrink, Yogo-Yogo) and other milk(product) drinks; chocolate-flavoured half fat / semi-skimmed milk; strawberries; white fish fillet, haddock, cod parings, fish fingers; eggs; boiled potatoes (including mashed potatoes) (hot meal); runner beans (hot meal); potato crisps, salted biscuits, Wokkels, Nibbits, other cocktail snacks; peanuts, coated peanuts, cocktail nuts; salad as fillings of toast or baguette; whipped cream; bratwurst, lettuce (hot meal); gravy derived from cooking fat; cutlet, pork steak, pork collops; thick cut of bacon (warm meal); coffee milk (liquid coffee creamer) in coffee; margarine from packet as cooking fat; sugar added to coffee, tea, natural yoghurt, muesli, cornflakes; margarine from tub as spread on bread, crackers, rusks, etc.

Negative loading for butter as cooking fat; butter as spread for bread; fennel; cheese (as part of a hot meal); brown bread, wholemeal bread (cold meal); tee; minced beef (hot meal); oil as cooking fat; full-cream, full-fat cheese (spread), Gouda, Edam; (unpolished rice); tomatoes (hot meal); olives as a snack; onions (hot meal); sweet pepper (hot meal); broccoli (hot meal); sour cream, crème fraiche; biscuits, cookies.

**Component 3, “raw vegetables salad”**

Positive loadings for dressing with raw vegetables salad; cucumber in raw vegetables salad; lettuce (cabbage, iceberg) in raw vegetables salad; carrots in raw vegetables salad; other vegetables in raw vegetables salad; lambs lettuce, purslane, water cress in raw vegetables salad; cabbage (white, Chinese, etc.) in raw vegetables salad; other vegetables (hot meal); sprouts (e.g., garden cress, mung bean sprouts (taugé), alfalfa) in raw vegetables salad; tomatoes (hot meal); strawberries; other types of fresh fruit; olives as a snack; onions (hot meal).

Negative loadings for: curly kale (hot meal); sauerkraut cabbage (hot meal); tangerines (mandarins); potatoes (including mashed potatoes) (hot meal); Brussels sprouts (hot meal); smoked sausage (hot meal).

**Component 4, “fish”**

Positive loadings for fennel; soup as a main course; raisin bread, currant bread, muesli bread (cold meal); oil as cooking fat; tomatoes (hot meal); other type of fish with dinner (hot meal); other types of fish; fried / deep-fried fish with dinner (hot meal); salmon steak/fillet; canned fish; mackerel, eel (fresh/raw or boiled, cured, steamed); olives as a snack; white fish fillet, haddock, cod parings, fish fingers; herring haring; onions (hot meal); sweet pepper (hot meal);

mussels; wine, rosé wine, sherry, port(-wine), vermouth, etc.; grapefruits (including freshly squeezed; lamb meat, mutton; beer; grapefruit juice ready-made (pack, bottle); eggs; porridge with low fat / skimmed (Brinta, oatmeal, semolina, rice, etc.); sour cream, crème fraiche; salads (egg salad, salmon salad, celery salad, etc.).

Negative loadings for light margarine as spread on bread, crackers, rusks, etc.; minced meat hot dog, croquette, or comparable snacks with hot meal; minced beef/pork (hot meal); chocolate confetti, chocolate spread, hazelnut spread.

**Component 5, “cooked vegetables”**

Positive loadings for brown bread, wholemeal bread (cold meal); oil as cooking fat; (unpolished rice); curly kale (hot meal); sauerkraut cabbage (hot meal); French beans (hot meal); cauliflower (hot meal); broccoli (hot meal); spinach (hot meal); carrots (hot meal); chicory (hot meal); leek (hot meal); boiled potatoes (including mashed potatoes) (hot meal); red cabbage (hot meal); endive (hot meal); runner beans (hot meal); Brussels sprouts (hot meal); bread beans (hot meal); kohlrabi (hot meal); low fat / skimmed yoghurt, biogarde, vifit, etc. without fruit (natural); pasta (macaroni, spaghetti, etc.); cutlet, pork steak, pork collops.

**Component 6, “Italian-like kitchen and sweet”**

Positive loadings: cheese (as part of a hot meal); nut spread, sesame spread; French fries / chips, with hot meal; salad cream, mayonnaise, etc. (not with raw vegetables); fried potatoes (hot meal); minced beef (hot meal); oil as cooking fat; full-cream, full-fat cheese (spread), Gouda, Edam; ice cream (not lemonade-ice); ready-made pasta meals (macaroni, spaghetti, lasagne, etc.); Chinese noodles (bami), fried rice (nasi), Chinese/Indian food (ready meals); olives as a snack; onions (hot meal); sweet pepper (hot meal) paprika; eggs; leek (hot meal); pasta (macaroni, spaghetti, etc.); red sauces (tomato ketchup, tomato sauce, etc.); sweets (liquorice, boiled sweets, peppermint, etc.); peanut sauce; potato crisps, salted biscuits, Wokkels, Nibbits, other cocktail snacks; ready-made oriental sauce; sour cream, crème fraiche; peanuts, coated peanuts, cocktail nuts; other hot sauces (from packet or self-made); chocolates, chocolate pralines; cake, tart, waffle (treacle waffle, almond paste filled tarts, etc.); biscuits, cookies; salads (egg salad, salmon salad, celery salad, etc.); salad as fillings of toast or baguette; pizza (hot meal); chocolate bars (Mars, Nuts, etc.); cheese as fillings of toast or baguette; whipped cream; cake, pie, pastry, filled flan; cheese cubes, cheese as a snack.

Negative loadings for boiled potatoes (including mashed potatoes) (hot meal).

**Component 7, “meat”**

Positive loadings for bratwurst, lettuce (hot meal); pork tenderloin, fricandeau, schnitzel; whole milk / full fat yoghurt, biogarde, vifit, etc. without fruit (natural); salami sausage, saveloy, luncheon meat (cold meal); loaf fried minced meat (cold meal); brown bread; canned fruit (apple sauce, rhubarb sauce, pears, etc.); solid frying fat as cooking fat; minced beef/pork (hot meal); other type of porridge, vanilla custard, pudding; curly kale (hot meal); sauerkraut cabbage (hot meal); boiled potatoes (including mashed potatoes) (hot meal); red cabbage (hot meal); bratwurst, lettuce (hot meal); coffee with caffeine; boiled ham, fricandeau, cured pork side (cold meal); gravy derived from cooking fat; cutlet, pork steak, pork collops; thick cut of bacon; boiled ham, fricandeau, cured pork side (cold meal); smoked sausage; smoked raw ham (cold meal); streaked beefsteak, ribs, stewing steak; coffee milk (liquid coffee creamer) in coffee; rashers streaky bacon, bacon (cold meal); margarine from packet as cooking fat; sugar added to coffee, tea, natural yoghurt, muesli, cornflakes; beef steak, baked and roast steak, roast beef, (raw) minced/ground steak; smoke-dried, roast beef, corned beef (cold meal); luncheon meat sausage, salami, cooked sausage as a snack; margarine from tub as spread on bread, crackers, rusks, etc.

Negative loadings for tofu, tempeh, soya chunks; vegetarian burger/schnitzel; vegetarian burger/schnitzel; quorn (meat substitute), small pieces / minced; quorn (meat substitute), burger/schnitzel; vegetarian processed meat products.

**Supplementary material 6** Comparison of the loading factors corresponding to the main food pattern components as identified by principal component analysis in participants with and without gestational diabetes (n=2803; expressed as mean ± SD.

|  | **With gestational diabetes**  **(n=37)** | **Without diabetes gestational**  **(n=2766)** | **p-value**  **(ANOVA)** |
| --- | --- | --- | --- |
| **Component** | mean ± SD | mean ± SD |  |
| **1, “lacto-ovo-vegetarian”** | -0.3554 ± 0.7390 | 0.0056 ± 1.0026 | **0.03** |
| **2, “fast food”** | -0.0322 ± 0.9814 | 0.0007 ± 1.0010 | 0.84 |
| **3, “raw vegetables salad”** | 0.2407 ± 0.8914 | -0.0031 ± 1.0014 | 0.14 |
| **4, “fish”** | 0.1192 ± 0.9464 | -0.0020 ± 0.9993 | 0.46 |
| **5, “cooked vegetables”** | 0.0270 ± 1.2056 | 0.0006 ± 0.9964 | 0.87 |
| **6, “Italian-like kitchen and sweet”** | -0.4341 ± 0.7549 | 0.0059 ± 1.0021 | **0.01** |
| **7, “meat”** | 0.2686 ± 0.9881 | -0.0025 ± 0.9999 | 0.10 |
